# Gene Expression and Protein Abundance of Nuclear Receptors in Human Intestine and Liver: A New Application for Mass Spectrometry-Based Targeted Proteomics

**DOI:** 10.3390/molecules27144629

**Published:** 2022-07-20

**Authors:** Christoph Wenzel, Lisa Gödtke, Anne Reichstein, Markus Keiser, Diana Busch, Marek Drozdzik, Stefan Oswald

**Affiliations:** 1 Department of Pharmacology, Center of Drug Absorption and Transport, University Medicine Greifswald, 17475 Greifswald, Germany; christoph.wenzel@med.uni-greifswald.de (C.W.); lisa.godke@med.uni-greifswald.de (L.G.); anne.reichstein@med.uni-greifswald.de (A.R.); markus.keiser@web.de (M.K.); diana.busch@med.uni-greifswald.de (D.B.); 2 Department of Experimental and Clinical Pharmacology, Pomeranian Medical University, 70-204 Szczecin, Poland; 3 Institute of Pharmacology and Toxicology, Rostock University Medical Center, 18057 Rostock, Germany

**Keywords:** nuclear receptors, intestine, liver, human, drug-drug interaction, enzymes, transporters

## Abstract

Background: Unwanted drug-drug interactions (DDIs), as caused by the upregulation of clinically relevant drug metabolizing enzymes and transporter proteins in intestine and liver, have the potential to threaten the therapeutic efficacy and safety of drugs. The molecular mechanism of this undesired but frequently occurring scenario of polypharmacy is based on the activation of nuclear receptors such as the pregnane X receptor (PXR) or the constitutive androstane receptor (CAR) by perpetrator agents such as rifampin, phenytoin or St. John’s wort. However, the expression pattern of nuclear receptors in human intestine and liver remains uncertain, which makes it difficult to predict the extent of potential DDIs. Thus, it was the aim of this study to characterize the gene expression and protein abundance of clinically relevant nuclear receptors, i.e., the aryl hydrocarbon receptor (AhR), CAR, farnesoid X receptor (FXR), glucocorticoid receptor (GR), hepatocyte nuclear factor 4 alpha (HNF4α), PXR and small heterodimer partner (SHP), in the aforementioned organs. Methods: Gene expression analysis was performed by quantitative real-time PCR of jejunal, ileal, colonic and liver samples from eight human subjects. In parallel, a targeted proteomic method was developed and validated in order to determine the respective protein amounts of nuclear receptors in human intestinal and liver samples. The LC-MS/MS method was validated according to the current bioanalytical guidelines and met the criteria regarding linearity (0.1–50 nmol/L), within-day and between-day accuracy and precision, as well as the stability criteria. Results: The developed method was successfully validated and applied to determine the abundance of nuclear receptors in human intestinal and liver samples. Gene expression and protein abundance data demonstrated marked differences in human intestine and liver. On the protein level, only AhR and HNF4α could be detected in gut and liver, which corresponds to their highest gene expression. In transfected cell lines, PXR and CAR could be quantified. Conclusions: The substantially different expression pattern of nuclear receptors in human intestinal and liver tissue may explain the different extent of unwanted DDIs in the dependence on the administration route of drugs.

## 1. Introduction

Human drug metabolism and transport are well accepted key determinants of the pharmacokinetics of many drugs and, in turn, of their efficacy and safety. There is a large body of evidence demonstrating a tremendous variability in gene expression and/or protein abundance of drug metabolizing enzymes (DMEs) and drug transporters in pharmacokinetically and highly relevant tissues including the intestine, liver and kidney [1,2,3]. The background of the observed high inter-subject variability in the expression and function of DMEs and transporter proteins may include extensively studied genetic polymorphisms [3,4], but also less investigated aspects such as environmental impacts on transcriptional and epigenetic regulation [5,6,7], post-translational modifications [8] or disease-related changes [9,10]. In order to estimate or predict the impact of those individual factors on the expression and function of DMEs and drug transporters, a deeper understanding of the respective regulatory mechanisms in different organs is required.

In this regard, nuclear receptors represent, thus far, the best established mechanism of regulation [11,12]. These receptors act as transcription factors, i.e., binding of endogenous or exogenous molecules (e.g., drugs) to nuclear receptors, resulting in dimerization with another nuclear receptor (mostly RXR), and subsequent binding of the complex to specific DNA sequences (via receptor-specific DNA-binding domains), which finally initiates the process of gene transcription. As several nuclear receptors are involved in the regulation of DMEs and drug transporters, activation of these receptors results in markedly increased expression and function of the respective DMEs and drug transporters [13]. Thus, exposure to nuclear receptor ligands such as rifampicin, St. John’s wort or carbamazepine, together with DMEs and transporter substrates, can result in unwanted drug–drug interactions (DDIs) due to diminished systemic or tissue drug exposure, which may threaten the intended therapeutic effect [14,15]. Associated to this, the nuclear receptors, pregnane X receptor (PXR) and constitutive androstane receptor (CAR), were especially reported to be of high clinical relevance [11,12]. The activation of different nuclear receptors is associated with a typical induction pattern; i.e., PXR is known to regulate, for example, cytochrome P450 (CYP) 3A4, CYP2C9, uridine diphosphate glucuronosyltransferase 1A1 (UGT1A1), P-glycoprotein (P-gp) and multidrug resistance-associated protein 2 (MRP2), whereas CAR controls the expression of CYP2B6, CYP2C19, breast cancer resistance protein (BCRP) and MRP4 [11,16]. There seems to be also a considerable regulatory overlap between PXR and CAR. However, our current knowledge is rather limited because the majority of the regulatory information was derived from various in vitro models [12].

A few studies demonstrating direct in vivo evidence for the regulation of human DMEs and drug transporters have been conducted in human intestinal tissue after oral administration of PXR ligands [17,18]. In these studies, nuclear receptor-mediated upregulation of intestinal drug transporters (e.g., P-gp, MRP2) or DMEs (e.g., CYP3A4, UGT1A1) resulted in markedly diminished plasma exposure of co-administered victim drugs. In addition to these rather experimental studies in healthy volunteers, this mechanism was also shown to cause dramatic interaction scenarios in clinical practice [14,19,20]. Until now, only very little was known about the expression pattern of nuclear receptors in different tissues, which makes it difficult to estimate an extent of potential DDIs in the dependence on the used administration route of perpetrator and victim drugs [21]. The same is true for the distinct tissue concentrations of ligands required for a pronounced activation of the nuclear receptors. The available expression data indicate substantial expression differences between tissues, which may translate to differences in induction potential of prototypical inducers in different organs, related to their binding affinity to different nuclear receptors and the respective expression profile of nuclear receptors [18,22]. However, available expression data are exclusively based on mRNA data, which may not necessarily correlate to the encoded proteins, i.e., the nuclear receptors [21]. Thus, it was the aim of this study to characterize the gene expression and protein abundance of clinically relevant nuclear receptors in human intestine and liver.

## 2. Results and Discussion

There is evidence from gene expression studies that there are tissue-specific nuclear receptor expression profiles, suggesting profound differences in the induction potential of nuclear receptor ligands in different organs [21,22,23]. In addition, several clinical DDI studies indicate that the extent of interaction, i.e., reduction of systemic drug exposure of victim drugs, is markedly affected by the route of administration of the perpetrator drug (e.g., rifampicin). In this regard, the oral administration of nuclear receptor ligands and the victim drug (substrates of CYP3A4 and/or P-gp) caused strikingly lower plasma levels (i.e., higher degree of interaction) compared to the intravenous administration of the victim drug [18,24,25,26,27,28]. Finally, some nuclear receptor ligands such as efavirenz were shown to induce only hepatic, but not intestinal, DMEs and drug transporters [29,30]. Therefore, we investigated in this study the gene expression and protein abundance of the clinically relevant nuclear receptors CAR, FXR, GR, HNF4α, PXR and SHP. A targeted proteomic method for absolute protein quantification was developed in order to provide reliable protein abundance data.

### 2.1. Assay Characteristics

The protein specificity of all predicted and finally used peptides was confirmed during method development by protein BLAST analysis. In order to develop a reliable quantification method, peptides with unfavorable features such as oxidative instability (due to cysteine, methionine, tryptophan or *N*-terminal glutamine), genetic polymorphisms and post-translational modifications were excluded. Finally, all used peptides and mass transitions applied for protein quantification were confirmed by wet-lab experiments.

For all peptides, stable isotope-labeled internal standards were used with a distinct mass difference of 8–10 Da (Table 1). All peptides were measured in the positive ionization mode using electrospray ionization, resulting, in each case, in doubly charged molecule ions. The observed *m*/*z* ratios of these ions were used as the Q1 filter setting. For all identified parent ions, manual product ion scans were performed to identify the three fragment ions with the highest intensity. After selecting the respective Q3 *m*/*z* ratios, the individual *m*/*z* transitions were manually optimized with respect to collision energy and declustering potential. The optimized mass spectrometer parameters are given in Table 1.

To the best of our knowledge, this is the first report on the LC-MS/MS-based quantification of nuclear receptors. Thus, our experimental details cannot be compared with other methods. As a potential limitation, our method used, in each case, only one proteospecific peptide for protein quantification (due to the high costs of quantitative peptides), which may bear the risk of obtaining misleading results due to different isoforms, incomplete protein digestion or truncated forms of the protein. However, the used peptides were also successfully identified by the Institute for Systems Biology (Seattle, WA, USA) as reported in their online repositories, i.e., the PeptideAtlas (http://www.peptideatlas.org, accessed on 20 June 2022) and the SRMAtlas (http://www.mrmatlas.org, accessed on 20 June 2022).

**Figure 1:**
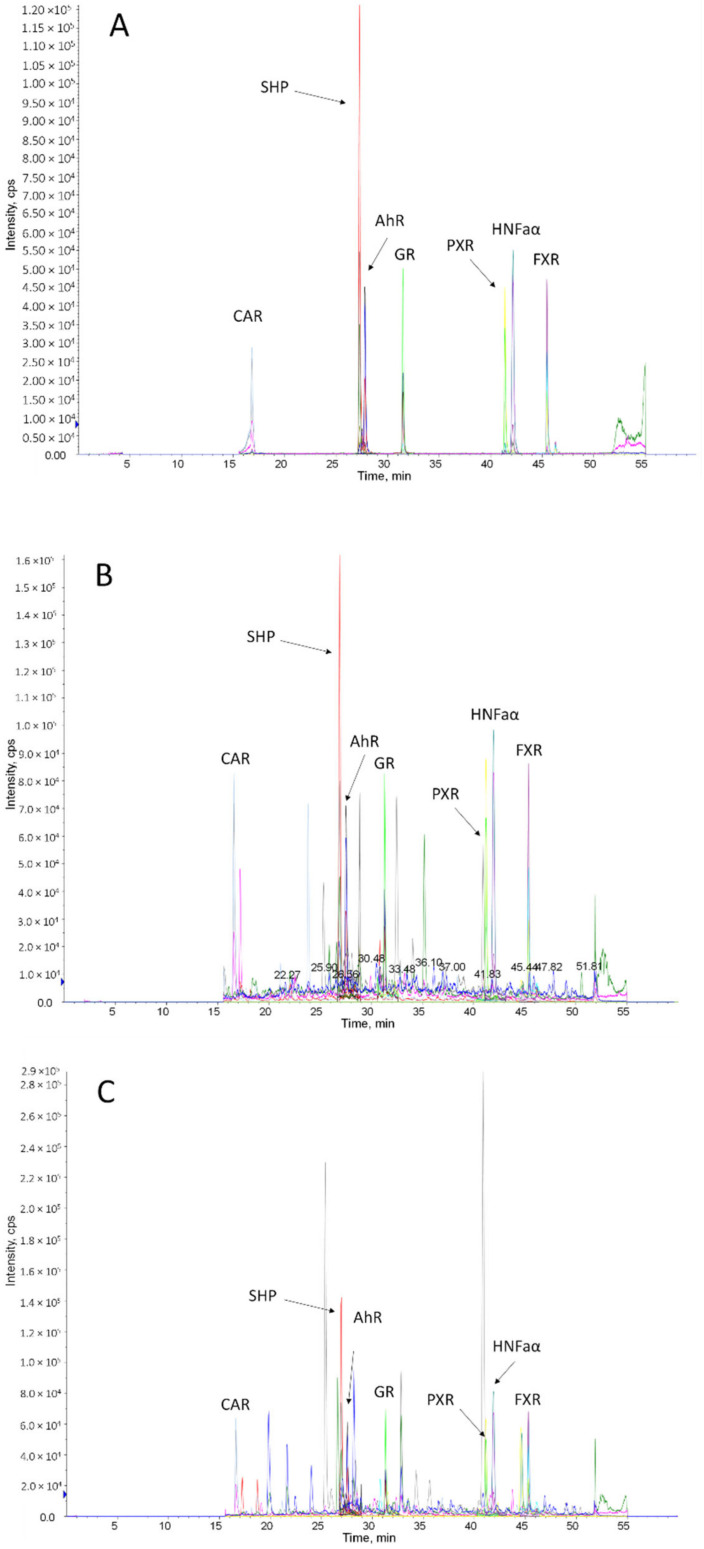
Total ion chromatogram of a digested HSA (validation matrix) sample (**A**), a human jejunum (**B**) and a human liver sample (**C**) spiked with internal standard peptides for all investigated nuclear receptors (each 5 nmol/L). Annotations indicate the respective nuclear receptor peptide.

To avoid chromatographic interferences of the individual peptides with each other and the complex biological matrix, the chromatography was performed using a 60-min gradient elution method. The peptides were chromatographically separated from each other and all included mass transitions for each peptide, where the internal standard peptides demonstrated co-elution (Figure 1). To assure optimal mass spectrometric detection and quantification, the dwell time was dynamically adapted to a fixed cycle time of 0.6 s using the scheduled MRM algorithm by the Analyst software that monitored altogether 30 *m*/*z* transitions (Table 1).

### 2.2. Method Validation

Our method validation was mostly based on the current bioanalytical method validation guidelines from the FDA and EMA [31]. In this regard, we validated the method for specificity, linearity, within-day and between-day accuracy and precision, as well as for matrix effects and stability. As a classical blank matrix was not available (nuclear receptors are present in nearly all cells), digested HSA (2 mg/mL) was used as the blank matrix for all validation procedures. Although this matrix is far off from being as complex as digested human tissue, it may mimic at least one digested protein of human origin and have the same total protein concentration (2 mg/mL) as used in our tryptic digested samples.

The developed method was found to be selective for the determination of all proteospecific peptides in digested has, as well as those in digested human intestinal and liver tissue, as concluded from the absence of analytical signals in different blank matrix samples and chromatographic or mass spectrometric interferences between the analytes, the internal standards and the biological matrix (Figure 1).

For all peptides, a linear correlation between peptide concentration and the analytical signal over the entire analytical range (0.1–50 nmol/L) was observed. The resulting correlation coefficients (r) for all calibration curves and peptides ranged between 0.9976–0.9999 (in each case N = 6) (Table 2).

The lower limit of quantification (LLOQ) of our quantitative assays was 0.1 nmol/L (1.5 fmol on column) for all investigated nuclear receptors. Here, the analytical signal was at least >5 times above that of the respective blank matrix samples. The sensitivity of our method is comparable to other targeted LC-MS/MS methods for DMEs and drug transporters [32,33,34,35,36]. Within-day (intra-day) as well as between-day (inter-day) accuracy and precision in the validation matrix were within the range of ±15% of the nominal concentrations and <15% for the respective coefficients of variation (CV) of the mean values for all peptides (Table 2).

All peptides demonstrated sufficient stability (±15% of the initial concentrations at low, medium and high concentrations) during storage in the cooled autosampler rack for 24 h and during up to three freeze–thaw cycles (Table 3).

The classical investigation of matrix effects could not be achieved in our study due to the lack of availability of a blank, free-of-endogenous nuclear receptor matrix for human tissue lysates. However, our validation matrix was shown to have no impact on the accuracy of our quantitative method compared to the quality control samples without any matrix (Table 3). This is most likely due to our long gradient elution time combined with high resolution chromatography and the use of stable isotope-labeled internal standard peptides which compensate for ion suppression or enhancement effects as caused by the biological matrix.

### 2.3. Application of the Method

The developed and validated method was applied to quantify the protein amounts of clinically relevant nuclear receptors in human tissue samples. Here, human liver, jejunum, ileum and colon samples from, in each case, eight donors have been analyzed (inter-subject comparison). In parallel, the respective mRNA expression was studied.

**Figure 2:**
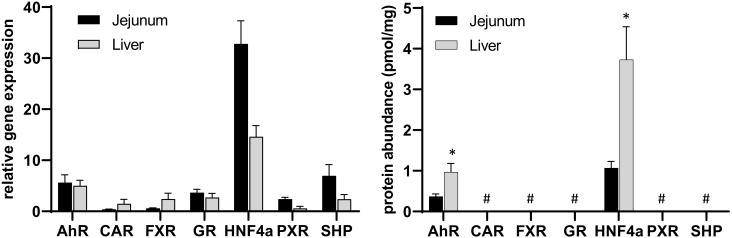
Data on gene expression (**left**) and protein abundance (**right**) of the investigated nuclear receptors in human jejunum and liver where, in each case, N = 8 different donors (inter-subject comparison). Data given as mean ± SD. *: *p* ≤ 0.05 compared to jejunum, #: < lower limit of quantification.

As shown in Figure 2, the expression pattern of nuclear receptors differed markedly within the liver and jejunum, which is the most relevant intestinal section for intestinal drug absorption.

Associated to this, gene expression data demonstrated a significantly higher expression of PXR, HNF4α and SHP in the jejunum than in the liver, whereas CAR and FXR were found to have a significantly higher expression in human livers (Figure 2, left panel). With the exception of AhR and HNF4α, all other nuclear receptor abundances were below the lower limit of quantification (Figure 2, right panel). In contrast to the gene expression data, the protein abundance of HNF4α and AhR were significantly higher in the liver than in the jejunum, which underlines the known fact that gene expression data do not necessarily correlate well with the encoded protein level. Interestingly, HNF4α and AhR were also shown to be highly expressed on the mRNA level so that the lack of protein detection for the other investigated nuclear receptors fits to the lower transcriptional processing of the respective genes. The low or undetectable protein abundance may be explained by the mode of action of nuclear (hormone) receptors, which can be activated by very low ligand concentrations and act in a repetitive manner [11,15,16]. Thus, classical dose-response relationships may not apply for nuclear receptors.

The reason that we were able to quantify in a robust manner only two of our seven proteins of interest, in human intestine and liver, is most likely due to the limited sensitivity of the mass spectrometer. Although we used a high-sensitivity instrument (QTRAP 5500), this was not sensitive enough to quantify low-abundant nuclear hormone receptors in a complex biological matrix. In line with our observation, we are not aware of any study showing protein data of nuclear receptors independent from the analytical method (Western blotting, global or targeted proteomics) in human tissue. Another reason might be related to the chosen peptides, because different peptides may result in slightly different sensitivities to the method. However, due to economic reasons, we were not able to analyze multiple peptides for each nuclear receptor. In our selection process, we carefully chose the most promising peptides, which were also reported to be observable in proteomic databases (e.g., PeptideAtlas).

Based on our current knowledge, PXR seems to play an important role in the clinically relevant regulation of human intestinal drug transporters, whereas CAR and, to a lesser extent, PXR appear to be more relevant for DDIs in the liver [11,12,16]. Hence, the route of drug administration (i.e., oral vs. intravenous) can have a profound impact on the extent of DDIs for drugs undergoing significant NR-mediated regulation (e.g., CYP3A4 metabolism and/or P-gp efflux [18,24,25,26,27,28], 2020). This conclusion from clinical studies is confirmed by our expression data, at least on an mRNA level (i.e., intestinal PXR > hepatic PXR; intestinal PXR >> intestinal CAR) [21,22,23].

**Figure 3:**
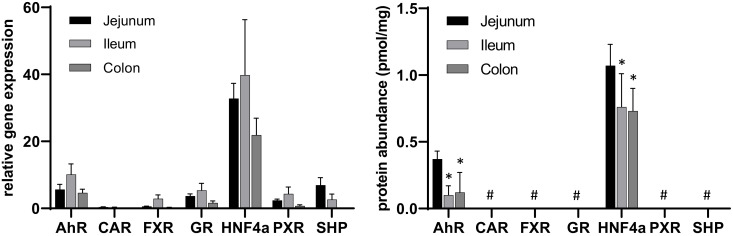
Data on gene expression (**left**) and protein abundance (**right**) of the investigated nuclear receptors along the human intestine (jejunum, ileum, colon) as measured, in each case, in N = 8 different donors (inter-subject comparison). Data given as mean ± SD. *: *p* ≤ 0.05 compared to jejunum, #: < lower limit of quantification.

In line with this conclusion, and also as seen in the human intestine, PXR has a strikingly higher expression than CAR (Figure 3, left panel), which was also confirmed in a previous analysis [23]. The rank order of mRNA expression in the different intestinal fractions is HNF4α > AhR > SHP > GR > PXR > FXR > CAR. Of the mentioned nuclear receptors, PXR and CAR especially (and partly FXR) are of great clinical importance as they regulate several highly important DMEs and drug transporters [11,12,15]. Comparable to the liver, only HNF4α and AhR could be detected on a protein level in all intestinal sections (Figure 3, left panel) (HNF4α > AhR).

**Figure 4:**
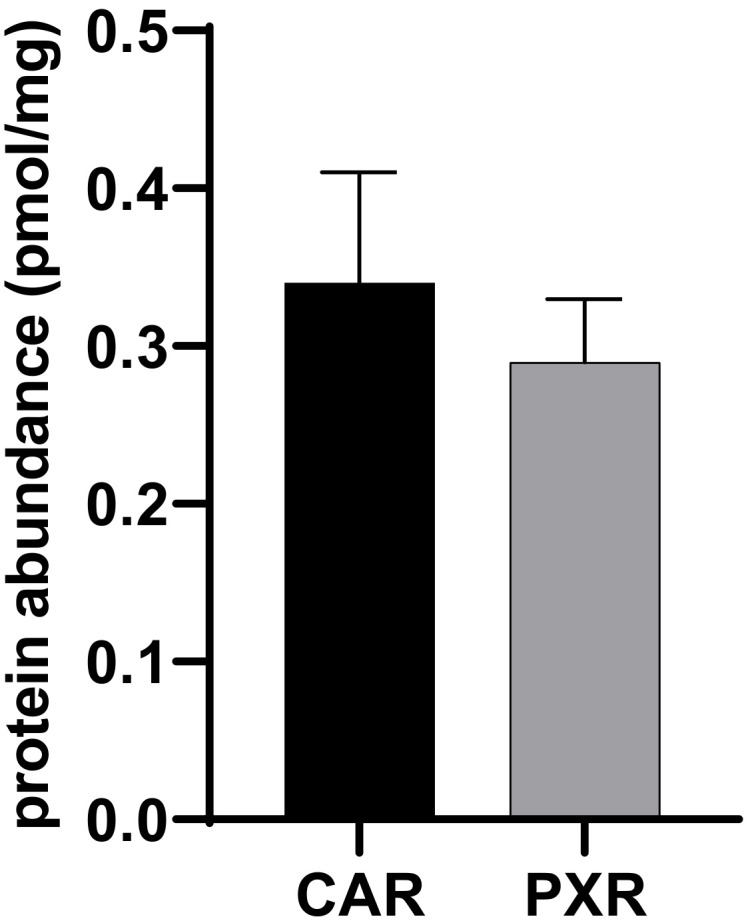
Protein abundance of CAR and PXR as measured in stably transfected MDCK-CAR and MDCKII-PXR cells. Data given as mean ± SD.

Considering the outstanding role of PXR and CAR in the regulation of DMEs and drug transporters, we generated stably transfected MDCKII cells overexpressing PXR and CAR to, finally, check the functionality of our method in biological samples. Both proteins could be successfully identified in MDCKII-CAR and MDCKII-PXR cells, which confirms the feasibility of PXR and CAR detection using our method (Figure 4).

## 3. Materials and Methods

### 3.1. Reagents and Consumables

LC-MS-grade acetonitrile (ACN) with 0.1% formic acid (FA) and LC-MS-grade water with 0.1% FA were purchased from Carl Roth (Karlsruhe, Germany). Deionized water (conductance: ≤0.055 μS/cm, pH 5.0–6.0) was generated with the Astacus system (membrapure, Hennigsdorf, Germany). Ethylenediaminetetraacetic acid (EDTA), human serum albumin (HSA), iodoacetamide (IAA), phosphate-buffered saline (PBS) and formic acid (FA) were purchased from Sigma-Aldrich (Steinheim, Germany). Ammonium bicarbonate (ABC), dithiothreitol (DTT), sodium dodecyl sulfate (SDS), sucrose and Tris-(hydroxymethyl)-aminomethane hydrochloride (Tris-HCl) were obtained from Carl Roth (Karlsruhe, Germany). The BCA kit to measure unspecific protein concentrations was from Thermo Fisher Scientific (Schwerte, Germany). Protease inhibitor cocktail III was ordered from Merck (Darmstadt, Germany). Custom-made peptide standards and the corresponding stable isotope-labeled internal standards were synthesized by JPT Peptide Technologies (Berlin, Germany) or Thermo Fisher Scientific. All peptides were of analytical grade (>95%), which was verified by exact quantification via amino acid analysis and certified by the respective manufacturers. Sequencing Grade Modified Trypsin and ProteaseMAX™ surfactant were purchased from Promega (Mannheim, Germany). Proteomic sample preparation was conducted in Protein LoBind Tubes (Eppendorf, Hamburg, Germany).

### 3.2. Intestinal Tissue

Intestinal tissue from the jejunum, ileum and colon, as well as liver tissue, was collected from, in each case, 8 different patients undergoing surgery for different medical reasons. Supplementary Table S1 gives an overview about the patient characteristics. All tissue samples were free of macroscopic signs of inflammation or necrosis as assessed by an experienced visceral surgeon. The collected samples were immediately snap frozen in liquid nitrogen and grinded in a stainless steel mortar to be finally stored at −80 °C until further analysis. The study was approved by the local Ethics Committee of the University of Medicine, Greifswald.

### 3.3. Gene Expression Analysis

For isolation of total RNA, approximately 30 mg of each frozen tissue powder was used for extraction with the NucleoSpin^®^ miRNA Kit (Macherey-Nagel, Düren, Germany). Quantity and purity of isolated RNA was determined by using a NanoDrop ND-1000 spectrophotometer (NanoDrop Technologies, Wilmington, DE, USA).

Quality and integrity of RNA samples was assured using the Agilent^®^ 2100 Bioanalyzer^®^ (Agilent Technologies, Waldbronn, Germany) and was stated as an RNA integrity number (RIN) ranging from 6.6 to 9.0 (Supplementary Table S1). cDNA was prepared from 2 µg of total RNA using the High-Capacity cDNA Reverse Transcription Kit (Applied Biosystems, Darmstadt, Germany) according to the manufacturer’s instructions. The gene expression levels of AhR, NR1I3 (CAR), NR1H4 (FXR), NR3C1 (GR), HNF4A (HNF4α), NR1I2 (PXR) and NR0B2 (SHP) were examined by quantitative real-time PCR analysis using TaqMan^®^ Gene Expression Assays (Applied Biosystems) following the instructions of the manufacturer on MicroAmp^®^ Optical 96-Well Reaction Plates. Table 4 provides an overview about the used predeveloped gene expression assays. Each sample was analyzed with a 7900HT Sequence Detection System (Applied Biosystems) simultaneously in two technical replicates; mean Ct (cycles of threshold) values were used for further analysis. Gene expression was calculated as the relative expression to the endogenous reference genes 18S rRNA, GAPDH (glyceraldehyde 3-phosphate dehydrogenase) and PGK1 (phosphoglycerate kinase 1) (2-ΔCt values).

Isolation of the nuclear fraction: Approximately 300 mg of frozen tissue powder was placed in prechilled Douncers and homogenized with 1 mL of lysis buffer (0.2% SDS, 5 mmol/L EDTA) containing 5 µg/mL of protease inhibitor solution. Homogenates were placed in Protein LoBind tubes on a vertical shaker for 30 min at 40 rpm and 4 °C, and afterwards, centrifuged for 5 min at 600× *g* and 4 °C. Subsequently, the supernatant (cytosolic fraction) was discarded and the resulting pellet containing membrane-bound proteins and the nuclear fraction was suspended in 300 µL of resuspension buffer (0.25 mol/L sucrose, 1 mmol/L EDTA in distilled water at pH 7.4) containing 5 µg/mL of protease inhibitor solution and stored at 80 °C until analysis.

### 3.4. Protein Quantification by LC-MS/MS Analysis

#### 3.4.1. Identification of Proteotypic Peptides

Proteospecific peptides for CAR, FXR, GR, HNF4α, PXR and SHP were identified by using a combined approach of in silico predictions and experimental data as described elsewhere [37]. In brief, the respective protein sequences (database: UniProtKB/Swiss-Prot, https://legacy.uniprot.org/uniprot/, accessed on 20 June 2022) were subjected to an in silico trypsin digestion (https://web.expasy.org/peptide_mass/, accessed on 20 June 2022), allowing a sequence length of 7–20 amino acids and excluding any missed cleavages sites. Furthermore, peptides with the following features were excluded: 1. cysteine, methionine or tryptophan (prone to oxidation), 2. amino-terminal glutamine (to avoid in-source cyclization to pyroglutamate), 3. non-synonymous genetic polymorphisms (allele frequency >1%), 4. experimentally proven post-translational modifications (altered mass), and 5. repeated sequences of arginine and lysine (risk of missed cleavage by trypsin). Finally, the protein specificity of each observed peptide was assured by an NCBI protein BLAST search against the UniProtKB/Swiss-Prot database (https://blast.ncbi.nlm.nih.gov/, accessed on 20 June 2022).

The identified sequences were ordered as crude peptides (SpikeTides™, JPT Peptide Technologies, Berlin, Germany) to set up quantitative mass spectrometry methods and to identify the best peptides in terms of sensitivity and chromatographic properties. After identification of the best observable peptide for each protein, these peptides were ordered in unlabeled and stable isotope-labeled forms that were high-analytical-grade quality from Thermo Fisher Scientific. The appropriate mass transitions, collision energies and declustering potentials for each proteospecific peptide were identified and optimized by manual infusion to the tandem mass spectrometer QTRAP 5500 (Sciex, Darmstadt, Germany). For each peptide and the respective internal standard peptide, three mass transitions of highest intensity were selected (Table 4).

#### 3.4.2. Sample Preparation and Digestion Procedure

The protein concentration of the isolated nuclear/membrane fraction from intestinal and hepatic tissue was determined with the BCA protein assay. The samples were adjusted to a protein concentration of 2 mg/mL with PBS. A total of 100 µL of each sample was mixed with 10 µL of DDT (200 mmol/L), 40 µL of ABC (50 mmol/L, pH 7.8) and 10 µL of ProteaseMax™ surfactant trypsin enhancer and incubated at 60 °C for 20 min (denaturation). After cooling down, 10 µL of IAA (400 mmol/L) was added and the samples were incubated for 15 min at 37 °C in the dark (alkylation). For protein digestion, trypsin was added with a trypsin/protein ratio of 1:40 and samples were incubated at 37 °C for 16 h. The digestion was stopped by adding FA (20 µL, 10% *v*/*v*). Finally, the samples were centrifuged for 10 min at 16,000× *g* and 4 °C, stable isotope-labeled internal standards were added to the clear supernatant (final concentration: 5 nmol/L) and samples were transferred into HPLC vials prior to LC-MS/MS analysis. All sample preparation and digestion steps were performed by using Protein LoBind Tubes (Eppendorf) to avoid sample loss.

#### 3.4.3. LC-MS/MS Analysis

LC-MS/MS analyses were conducted on a 5500 QTRAP triple quadrupole mass spectrometer (Sciex) coupled to an Agilent 1260 Infinity Binary HPLC system (Agilent Technologies, Waldbronn, Germany) controlled by the Analyst 1.6.3 software (Sciex). Chromatographic separation was performed on a Kinetex^®^ 2.6 µm C18 100 Å core-shell column (100 × 2.1 mm, Phenomenex, Aschaffenburg, Germany) with gradient elution using acetonitrile containing 0.1% formic acid (solvent A) and water containing 0.1% formic acid (solvent B). The flow rate of the mobile phase was 200 µL/min and injection volume was 15 µL. The gradient applied was as follows: 2% solvent A for 5 min, followed by a linear gradient from 2–25% solvent A over 35 min, then increase solvent A to 50% within 13 min, switching to 60% for 3 min before coming back to 2% solvent A within 9 min. Column oven temperature was set to 50 °C, whereas the autosampler temperature was adjusted to 4 °C. The mass spectrometer was equipped with an electrospray ionization (ESI) Turbo V™ Ion Source interface operated in positive mode using the following gas parameters: source temperature, 500 °C; ion spray voltage, 5500 V; curtain gas, 50 psi; ion source gas 1, 50 psi; ion source gas 2, 50 psi and high collision activated gas (all nitrogen). Mass spectrometry parameters such as declustering potential and collision energy were manually optimized for each single peptide, as mentioned above, and are summarized in Table 2.

#### 3.4.4. Preparation of Calibration Curves, Method Validation and Sample Measurements

For preparation of calibration curves and quality control (QC) samples, digested human serum albumin (HSA, 2 mg/mL) was used as the blank matrix and spiked with increasing amounts of each peptide to generate the following target concentrations: 0.1, 0.25, 0.5, 1.0, 2.5, 5, 10, 25 and 50 nmol/L (1.5–750 fmol on column) for calibration values and 0.5, 5 and 50 nmol/L for quality control (QC) samples. The stable isotope-labeled internal standard peptides were added to all samples (final concentration: 5 nmol/L).

Selectivity of the method was confirmed by analyzing six different batches of digested HSA; here, we compared the respective blank matrix samples with matrix containing either internal standard peptides, analytical peptides or both. Linearity was investigated by correlating the peak area ratios (analyte over the internal standard) with the spiked peptide concentrations and calculating calibration curves. Between-day (inter-day) accuracy and precision were evaluated by measuring six QC sample sets that were prepared and measured on different days. Within-day (intra-day) accuracy and precision were assessed by analyzing six QC sample sets prepared and measured on the same day. Accuracy was determined by calculating the relative error of the measured mean value compared to the nominal concentration, whereas precision represents the coefficient of variation of the measured values.

Stability was investigated by using, in each case, six QC sample sets. Post-preparative (rack) stability was assessed by measuring the samples immediately after preparation and after storing them in the cooled autosampler (4 °C) for 24 h. Freeze-thaw stability was studied by measuring the samples before and after, for up to three freeze-thaw cycles (storage at −80 °C). Stability was assumed if the peptide content after the given storage condition was within the acceptable range of accuracy, i.e., ±15%. Matrix effects were investigated by comparing six quality control sample sets prepared in the blank matrix (HSA, 2 mg/mL), as well as in diluted stock solutions without any matrix.

On each day of analysis, calibration curves and QC sample sets were freshly prepared as mentioned above. QC samples represented at least 5–10% of all analytical samples and were measured during the entire analytical run. The criterion of acceptance for an analytical run was if at least 4 of 6 of all measured QC samples were within a relative error range of ±15% at the LLOQ of the nominal values, as suggested by the current FDA/EMA guidelines on bioanalytical method validation.

### 3.5. Generation of MDCKII-CAR and -PXR

MDCKII cells were purchased from the European Collection of Cell Cultures (Salisbury, United Kingdom) and were grown in Dulbecco’s Modified Eagle Medium, supplemented with 10% fetal bovine serum, 4 mM/L glutamine, 100 units/ml penicillin, and 100 µg/ml streptomycin (PAA, Coelbe, Germany) at 37 °C, 95% humidity, and 5% CO_2_.

The coding sequence of the full-length CAR (GenBank accession no. NM_001077482.3) and PXR (GenBank accession no. NM_003889.4) cloned into the retroviral expression vector pQCXIN (Takara Bio Europe/Clontech, Saint-Germain-en-Laye, France) were purchased from Eurofins, Ebersberg, Germany. MDCKII cells were infected according to the instructions of the manufacturer and selected by 500 µg/ml of neomycin.

### 3.6. Statistical Analysis

For each surrogate peptide and the respective internal standard, three mass transitions were monitored. All chromatograms were evaluated with the MultiQuantTM 3.0.2 software (Sciex) using the internal standard method and peak area ratios for calculation of absolute protein amounts (linear regression, 1/x weighting). Final peptide concentrations represent mean values of three transitions for each peptide. The resulting protein amount (pmol/mg) was calculated by normalizing the specific nuclear receptor concertation (nmol/L) to the individually observed protein concentration (BCA assay, mg/mL).

All mRNA and protein expression data are presented as mean ± standard deviation. Statistical analysis was performed using the GraphPad Prism 9 Software (GraphPad Software, Inc., San Diego, CA, USA). Statistical significance of differences in mRNA and protein expression between the different intestinal sections and between liver and jejunum were evaluated using the non-parametric Mann-Whitney U test. P-values less than 0.05 were considered as significant.

## 4. Conclusions

We developed an LC-MS/MS method for the simultaneous quantification of the nuclear receptors AhR, CAR, FXR, GR, HNF4α, PXR and SHP. The developed and validated method fulfilled the requirements of current bioanalytical guidelines with respect to specificity, accuracy, precision, stability and matrix effects. The quantitative assay was successfully applied to measure the protein abundance in human tissue samples. However, due to the very low protein amounts, only HNF4α and AhR could be detected in in vivo samples, whereas PXR and CAR could be quantified in transfected and overexpressing cell lines. Despite the challenges of detectability of nuclear receptors, the information about their tissue distribution can prove to be useful for the understanding and estimation of unwanted DDIs.

## Figures and Tables

**Table 1:** Overview of used proteospecific peptides and mass spectrometry parameters for their detection in the positive multiple reaction monitoring mode (MRM). Dwell time was automatically optimized (scheduled MRM algorithm). CE, collision energy; DP, declustering potential; *m*/*z*, mass-to-charge ratio.

Analyte	Peptide	Mass Transitions (*m*/*z*)		CE [V]	DP [V]
		Q_1_	Q_3_		
AhR	NDFSGEVDFR	593.4	322.2	39	150
			809.5	27	150
			722.6	24	150
AhR *	NDFSGEVDF[R(13C6;15N4)]	598.2	332.2	39	150
			819.4	27	150
			732.4	24	150
CAR	AQQTPVQLSK	550.2	671.3	27	120
			900.5	23	120
			429.4	23	120
CAR *	AQQTPVQLS[K(13C6;15N2)]	554.1	679.4	27	120
			908.5	23	120
			429.1	23	120
FXR	LQEPLLDVLQK	648.5	371.3	27	190
			925.6	27	190
			388.4	39	190
FXR *	LQEPLLDVLQ[K(13C6;15N2)]	652.0	371.2	27	190
			933.6	27	190
			396.3	39	190
GR	LLEESIANLNR	635.9	787.4	31	170
			356.4	28	170
			485.2	26	170
GR *	LLEESIANLN[R(13C6;15N4)]	641.0	797.4	31	170
			356.2	28	170
			485.2	26	170
HNF4α	DVLLLGNDYIVPR	743.6	554.4	25	180
			647.5	45	180
			876.4	36	180
HNF4α *	DVLLLGNDYIVP[R(13C6;15N4)]	748.6	554.4	25	180
			657.3	45	180
			886.5	36	180
PXR	VVDQLQEQFAITLK	816.2	361.2	33	170
			474.4	35	170
			1077.4	36	170
PXR *	VVDQLQEQFAITL[K(13C6;15N2)]	820.3	369.2	33	170
			482.4	35	170
			1085.4	36	170
SHP	VLLTASTLK	472.8	620.4	20	130
			326.1	17	130
			448.2	32	130
SHP *	VLLTASTL[K(13C6;15N2)]	476.7	628.3	20	130
			326.2	17	130
			456.2	32	130

(*) Stable isotope-labeled peptide.

**Table 2:** Validation data of within-day and between-day accuracy and precision, as well as correlation coefficients, of the respective calibration curves for the simultaneous quantification of the nuclear receptor peptides. Validation range was 0.1–50 nmol/L and data were calculated from, in each case, six quality control samples sets (0.5, 5 and 50 nmol/L) measured on one day (within-day data) or on different days (between-day data). Accuracy is given as relative error of nominal concentrations and precision as coefficients of variation of mean concentrations. AhR, aryl hydrocarbon receptor; CAR, constitutive androstane receptor; FXR, farnesoid X receptor; GR, glucocorticoid receptor; HNF4α, hepatocyte nuclear factor 4α; PXR, pregnane X receptor; SHP, small heterodimer partner.

	Accuracy [%]		Precision [%]		Correlation Coefficient r
	Within-Day	Between-Day	Within-Day	Between-Day	
AhR	−1.7–13.2	−1.2–0.3	2.3–4.5	3.2–9.3	0.9984–0.9999
CAR	0.5–12.0	−2.3–(−0.8)	4.4–7.6	2.7–7.6	0.9976–0.9999
FXR	−1.8–6.3	−3.0–2.3	0.9–3.5	3.3–5.4	0.9992–0.9999
GR	−0.2–11.5	−2.1–3.6	1.4–4.1	3.0–5.8	0.9992–0.9999
HNF4α	−2.1–10.2	−2.3–3.2	0.8–3.7	3.8–7.3	0.9995–0.9998
PXR	−3.5–7.7	−2.1–6.5	2.1–6.8	4.3–5.5	0.9988–0.9999
SHP	−0.9–0.7	−2.5–1.2	0.9–5.9	3.7–5.9	0.9992–0.9998

**Table 3:** Mean data for matrix effects and stability as assessed by analyzing, in each case, six quality control sample sets. AhR, aryl hydrocarbon receptor; CAR, constitutive androstane receptor; FXR, farnesoid X receptor; GR, glucocorticoid receptor; HNF4α, hepatocyte nuclear factor 4α; PXR, pregnane X receptor; SHP, small heterodimer partner.

	Matrix Effect [%]	Rack Stability 24 h @ 4 °C [%]	Freeze–Thaw Stability [%]		
			1st Cycle	2nd Cycle	3rd Cycle
AhR	95.9–102.0	95.6–100.7	85.8–105.8	91.5–100.2	90.9–97.4
CAR	100.3–101.8	93.6–98.3	90.6–105.1	90.3–103.2	86.9–89.4
FXR	96.3–113.8	100.1–104.7	88.9–104.4	93.2–99.9	90.8–100.1
GR	91.2–101.5	97.1–102.9	90.8–106.6	92.9–102.1	95.1–99.5
HNF4α	88.5–95.8	98.2–104.3	87.6–104.9	91.7–100.7	89.3–99.1
PXR	82.8–91.3	99.8–104.6	86.6–98.5	91.0–95.8	93.3–93.6
SHP	97.2–99.3	93.4–100.8	92.3–104.8	93.7–99.5	93.3–98.5

**Table 4:** Overview of used gene expression assays and proteospecific peptides.

Protein (Alias)	Gene Name	TaqMan© Assay I.D.	Peptide
AhR (BHLHE76)	AhR	Hs00169233_m1	NDFSGEVDFR
CAR	NR1I3	Hs00901571_m1	AQQTPVQLSK
FXR (BAR)	NR1H4	Hs01026590_m1	LQEPLLDVLQK
GR (GCR)	NR3C1	Hs00353740_m1	LLEESIANLNR
HNF4α (HNF4, NR2A1)	HNF4A	Hs00230853_m1	DVLLLGNDYIVPR
PXR (BXR)	NR1I2	Hs01114267_m1	VVDQLQEQFAITLK
SHP (SHP1)	NR0B2	Hs00222677_m1	VLLTASTLK
**Reference gene(s)**			
18S	18S	Hs99999901_s1	-
GAPDH	GAPDH	Hs02758991_g1	-
PGK1	PGK1	Hs00943178_g1	-

AhR, aryl hydrocarbon receptor; CAR, constitutive androstane receptor; FXR, farnesoid x receptor; GAPDH, glyceraldehyde 3-phosphate dehydrogenase; GR, glucocorticoid receptor; HNF4α, hepatocyte nuclear factor 4 alpha; LXR, liver x receptor; PGK1, phosphoglycerate kinase 1; PXR, pregnane x receptor; SHP, small heterodimer partner.

## Supplementary Materials

The following supporting information can be downloaded at: https://www.mdpi.com/article/10.3390/molecules27144629/s1, Table S1: Overview of patient and sample characteristics.

## Sample Availability

Samples of the compounds are not available from the authors.
