# Supplementary material for: Gene Expression and Protein Abundance of Nuclear Receptors in Human Intestine and Liver: A New Application for Mass Spectrometry-Based Targeted Proteomics
Source: Molecules. 2022 Jul 20;27(14):4629. doi: 10.3390/molecules27144629 (PMC9318449; doi:10.3390/molecules27144629)
Supplement: Supplementary file 1 [file molecules-27-04629-s001.zip › molecules-1815267-supplementary.pdf]

Table S1. Overview of patient and sample characteristics.

	Jejunum	Ileum	Colon	Liver
**Sample number**	8	8	8	8
**Male/female**	2/6	4/4	4/4	6/2
**Age (mean)**	68.3	72.1	69.3	47.9
**Age (range)**	60–75	60–90	46–86	27–73
**RIN (mean)**	8.5 ± 0.4	8.2 ± 0.5	8.3 ± 0.6	7.6 ± 0.9

RIN, RNA integrity number.
